# A Digital Twin Approach for the Improvement of an Autonomous Mobile Robots (AMR’s) Operating Environment—A Case Study

**DOI:** 10.3390/s21237830

**Published:** 2021-11-25

**Authors:** Paweł Stączek, Jakub Pizoń, Wojciech Danilczuk, Arkadiusz Gola

**Affiliations:** 1 Department of Automation, Faculty of Mechanical Engineering, Lublin University of Technology, 20-618 Lublin, Poland; p.staczek@pollub.pl; 2 Department of Organization of Enterprise, Faculty of Management, Lublin University of Technology, 20-618 Lublin, Poland; j.pizon@pollub.pl; 3 Department of Production Computerisation and Robotisation, Faculty of Mechanical Engineering, Lublin University of Technology, 20-618 Lublin, Poland; danilczuk.wojciech@gmail.com

**Keywords:** Industry 4.0, IoT, digital twin, AMR (autonomous mobile robots), AGV (automated guided vehicles), computer simulation

## Abstract

The contemporary market creates a demand for continuous improvement of production, service, and management processes. Increasingly advanced IT technologies help designers to meet this demand, as they allow them to abandon classic design and design-testing methods in favor of techniques that do not require the use of real-life systems and thus significantly reduce the costs and time of implementing new solutions. This is particularly important when re-engineering production and logistics processes in existing production companies, where physical testing is often infeasible as it would require suspension of production for the testing period. In this article, we showed how the Digital Twin technology can be used to test the operating environment of an autonomous mobile robot (AMR). In particular, the concept of the Digital Twin was used to assess the correctness of the design assumptions adopted for the early phase of the implementation of an AMR vehicle in a company’s production hall. This was done by testing and improving the case of a selected intralogistics task in a potentially “problematic” part of the shop floor with narrow communication routes. Three test scenarios were analyzed. The results confirmed that the use of digital twins could accelerate the implementation of automated intralogistics systems and reduce its costs.

## 1. Introduction

Increasingly sophisticated IT system solutions open up new horizons for the development of technical measures applicable in diverse areas of the economy [1,2]. Advances in both computer hardware and computational technology allow the collection and analysis of large amounts of data [3]. This infrastructural-and-analytical perspective paves the way to mass digitization, which is a fundamental component of Industry 4.0 [4]. The term Industry 4.0, which alludes to the past industrial revolutions, stands for the newest revolution, which essentially involves the digitization of processes and their components [5]. Each of the past revolutions introduced a new technical solution that had a critical impact on the development of industry in a given era. The first three industrial revolutions were brought about by mechanization, electrification, and the use of information systems [6]. Currently, the introduction of technologies and solutions associated with the Internet of Things (IoT), Cloud Computing, and Cyber-Physical Systems drives the Fourth Industrial Revolution [7]. These technologies have particular applications in the digitization of manufacturing and logistics processes and current mapping, diagnosis, and analysis of those processes [8]. The important fact that nowadays, thanks to these technologies, analyses are available in near real-time, that is, directly at the time when the changes are taking place, allowing the right decision to be taken at the right time.

The more and more advanced digitization technology also affords a completely different approach to the problems of designing and improving production systems and processes, as it eliminates the costly stage of testing the design assumptions in real-life conditions [9]. On the other hand, digitalization requires the implementation of appropriate protection mechanisms as well as investments in IT infrastructure. Furthermore, it should be remembered that the quality of the results obtained through digitization depends on the quality of the data obtained—which should be the focus of all researchers. A special role in this respect is played by the Digital Twin technology, which permits us to represent a real-world object in a digital world and to approximate its past and present states and forecast its future states in order to control the decision-making process [10]. The aim of this study was to show how the Digital Twin technology could be applied in testing the operating environment of an autonomous mobile robot (AMR). In particular, the concept of the Digital Twin was used to test the design assumptions in an early phase of the implementation of an AMR vehicle in a production hall. This was carried out by testing and improving the case of a selected intralogistics task in a potentially “problematic” part of the shop floor with narrow communication routes.

This paper is divided into three main parts. The first part (Section 2) presents the concept of the Digital Twin technology and its main assumptions. It describes how digital twins work and how they can be deployed as well as the benefits of their application. Section 3 is devoted to the issues of the use of AMR vehicles in the industrial environment and the main problems related to testing the design assumptions for the operation of this type of vehicle in the difficult conditions created by the robot’s surroundings. Section 4 provides an example of a practical application of the Digital Twin technology in testing the assumptions for the implementation of an AMR on a shop floor of a company producing power drive components. The article ends with a Conclusions section, which summarizes the most important points of the present study and outlines the directions of future research in the area discussed in this paper.

## 2. The Fundamentals and Main Assumptions of the Digital Twins Technology

**Figure 1:**
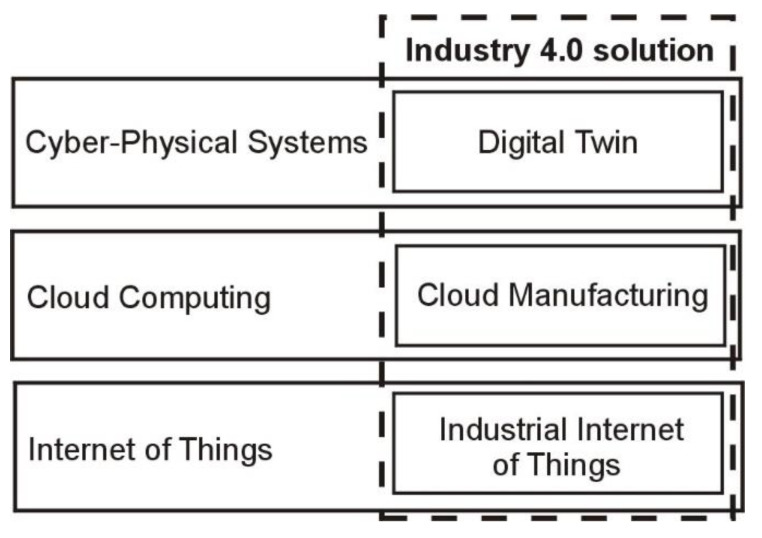
General scheme of Industry 4.0 solutions.

The development of Industry 4.0 systems is based mainly on IT solutions dedicated to production and in-house logistics [11]. These technologies constitute adaptations of native information technologies to industrial requirements and combine, in an interdisciplinary fashion, production engineering, IT systems, mechatronics, communication technologies, and data analytics [12]. The assumptions of Industry 4.0 are a set of functionalities of these information technologies, selected and combined as required by a production company’s business needs (Figure 1). As they are implemented in Industry 4.0, these technologies are given specialist names, such as Industrial Internet of Things, Cloud Manufacturing, or Digital Twin. Coupled together, they create Industry 4.0 solutions [13].

One way in which the diverse information technologies can be combined in an interdisciplinary manner is to create a digital twin solution. This issue is a new topic that has, in recent years, attracted the attention of numerous scientists. Importantly, it is also becoming popular among managers and practitioners, which shows that it is one of the most fertile and profitable areas of engineering and IT research [14]. The very term “digital twin” is defined in the literature in a multifarious manner. According to some authors, a digital twin is a virtual representation/model that interacts with a physical system throughout its life cycle [15]. Other widely known definitions relate to information exchange between two spaces that include sensors, data, and models [16]. Still others define the digital twin as the cyber part of a Cyber-Physical System (CPS) [17]. According to the definition proposed by the National Institute of Standards and Technology (NIST), “a digital twin is the electronic representation—the digital representation—of a real-world entity, concept or notion, either physical or perceived” [18]. Digital twins are deployed in areas such as optimization, monitoring, diagnosis, and prediction [19].

**Figure 2:**
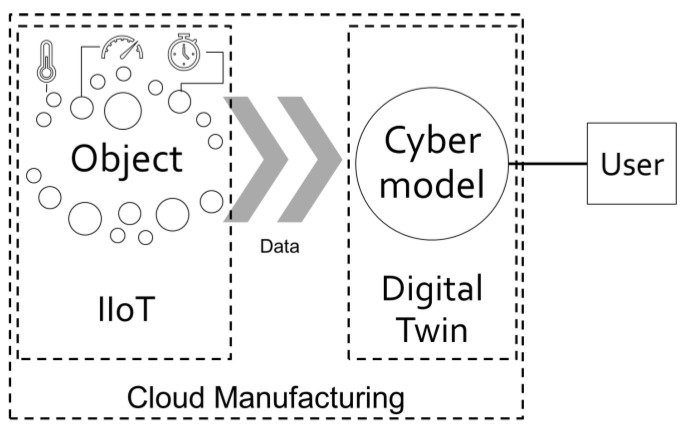
Digital twin data flow.

Digital twins consist of a physical part and a digital part (Figure 2). The physical part is the object/device/physical counterpart and the sensors with the embedded technologies are responsible for obtaining, aggregating, and transferring data. The digital part, on the other hand, involves technologies (hardware infrastructure, software) that allow for the creation of a digital module. The two parts exchange information collected via computer network links, with all calculations supported, for example, by a cloud computing model. Importantly, such systems have the benefit of allowing the user to operate at the level of the digital model rather than directly at the level of the physical device.

Depending on a specific need, the real-world object may be represented either very accurately or only approximately. How faithful a representation of the physical object a digital twin is depends both on how much information can be obtained about the object’s structure, its parameters, and cause, state, and effect variables, as well as on the business need (the purpose of using the model). In other words, Digital Twins can be simple or highly complex. When the second case happens, which often happens within the construction industry, for example, DT is often assumed as an SoS—System of Systems [2,20].

In engineering applications, models are mainly based on mathematical description of the laws of physics. However, for complex systems, “black-box” models are also used. Data-driven models are more and more commonly deployed to identify and describe the relationships between various variables [10].

The most important thing in the modeling process is to determine which of the model’s variables will be the key ones. Often, these variables are defined on the basis of the states that a given object is in. States refer to aspects of a system which evolve in time relative to their previous values and causal variables (for example, ones imposed externally by the system’s interactions with its environment). System state monitoring thus allows to trace the history of a system’s behavior over time. System parameters, in turn, describe how the inputs to the system affect states and how different states relate to one another in time. In many cases, system parameters remain constant or change slowly. When they do change, the variability makes testing the physical object difficult. The situation is completely different for a digital representation of the object, which can be tested multiple times for different values of parameters and causal variables.

It is worth noting that states and parameters for a particular digital twin representation are defined at a specific level of abstraction selected for the idealized representation. The representation includes information about the system itself, as well as its environment and the related processes. However, the system’s states and the parameters stored in the virtual representation of an object are not easily exchanged between physical reality and the virtual representation. This is because not all system states can be directly measured with the available measurement methods due to technological or economic constraints.

Therefore, often, digital twins have different representation levels, which specify how faithful the digital copy is to its physical counterpart. Many definitions indicate that the digital twin represents the highest possible level of model fidelity. However, the level of model fidelity relates directly to the level of abstraction selected for the virtual representation. It can be assumed that models with a higher fidelity ensure a better fit between the physical system and the virtual representation. At the same time, this assumption may not be feasible if the information (variables) that can be obtained from the system are not appropriate for the given level of model fidelity. Uncertainties or observation and modeling errors resulting from insufficient data can belie the potential benefits arising from the use of a higher fidelity model. Faulty data can lead to wrong decisions. Hence, one should make sure at the stage of creating a digital twin that the variables and parameters of the model have been properly validated and tested. Additionally, even if the required data could be collected, there still remain additional challenges associated with the high-fidelity model approach, which include data storage management and constraints on data transfer, computing power, and decision support time. For these reasons, a digital twin is assumed to be a virtual representation of a specific physical system. The fidelity of the representation depends mainly on the specific case of use, which is determined by a company’s particular business need [21]. It is worth mentioning that the digital twin can be built incrementally. That is, at first the basic model of the object’s operation is mapped, then new characteristics are added, and finally the full mapping in both operation and 3D form of the object is completed.

The basic need which motivates the use of a digital twin is to monitor and test a system that changes over time. Digital twins are used to improve the efficiency of machines, conveyors, and other devices. By tracking and modeling changes that take place over time in the virtual representation as a function of input variables to the system, one can approximate the past, present, and future states in order to control the decision process [10]. In this context, digital twins are also used to model systems and to collect and analyses information about processes and people, and can thus be employed to solve complex problems. This approach is used for many applications for both AMR and other engineering problems [20,22,23].

Simulations using digital twins can be performed alongside physical experiments. When conducted in combination, virtual and physical tests permit to verify both the models and the quality of the digital twin representation. This enables experts to create and calibrate models used in optimization applications, as discussed later in this article [24].

## 3. The Application of AMR in the Industrial Environment of Industry 4.0

One of the trends of the Third Industrial Revolution (Industry 3.0), naturally pursued in Industry 4.0, is the robotization of production processes. Industry 3.0 assumed that production processes would be subject to automation involving, among others, the use of industrial robots. The intended task of industrial robots is to take over some of the repetitive operations so far performed manually by humans [25]. In Industry 4.0, this idea has been taken further to the point where robots’ actions will no longer have to be programmed, but will be performed autonomously. The operator’s only responsibility will be to give commands to the robot, and the machine will decide on its own how to execute those commands [26].

**Figure 3:**
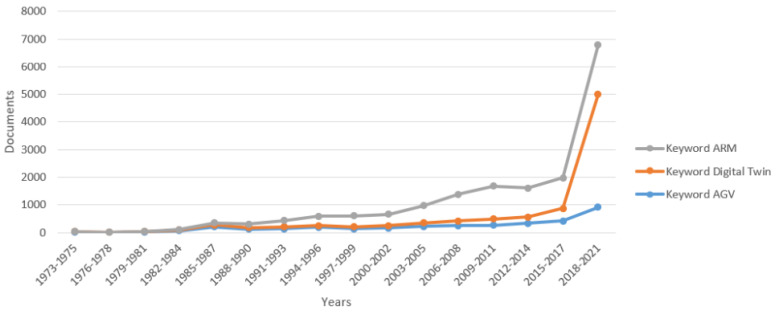
Number of publications regarding digital twins, AMR, and AGV according to the Scopus database.

The concept of automated robotic operations is realized by Automated Guided Vehicles (AGV) and AMR [27]. AMR are the latest solution in the sector of unmanned mobile robots for moving materials [28]. Most often, AMR are used in large warehouses or inside production plants to perform shop floor transport operations and handle materials, semi-finished products, finished products, or goods [29,30]. Statistics show that as early as 2000, about 20,000 autonomous vehicles were used for shop floor transport operations in industrial conditions [31]. In recent years, solutions of this type have become the focus of extensive research, and the number of scientific publications in this field is growing (Figure 3).

Based on practical experience related to the development and implementation of projects involving AMR solutions, we outline here the potential areas of application of the Digital Twins technology. We discuss an example of a practical use of a digital twin developed and deployed in an R&D project aimed at implementing an autonomous shop floor transport system. 

One area in which a digital twin can be constructed to support the implementation of an AMR is the development and testing of the robot’s navigation and localization algorithms. To create, improve, and optimize these algorithms, usually numerous tests of the real-world object have to be performed. In navigation and localization processes, the robot’s operating environment is also of key importance, since it determines how the robot’s localization algorithms and the autonomous AMR path planning algorithms operate [32]. In the case of mobile robots, the real-world objects to be considered include both the vehicle and the space in which it will be moving. Research and experiments on localization and navigation algorithms then require access to both the vehicle and its operating environment (or a laboratory emulating the operating environment). When prototyping such solutions, often, a research team has only one robot (prototype) to experiment with and a limited access to its target operating environment. In cases like that, building a digital twin seems to be the most beneficial solution. Vehicle control algorithms can be transferred to a virtual environment to allow research teams to work independently of whether or not they have access to the physical object. If a digital twin is used, several teams can work simultaneously on a solution, even if they only have one copy of the prototype. By recreating the robot’s operating environment as part of the digital twin, researchers can test the localization and navigation algorithms in the virtual operating environment when the real shop floor cannot be used (e.g., because the experiments would disrupt the production processes taking place in the factory). It may also be easier to introduce changes and test new variants of control algorithms in the environment of the digital twin than in the real-world setting. For example, if one wants to test the operation of a navigation algorithm under disruptions (e.g., illegible navigation markers, sensor damage), one can conduct an experiment in the digital environment and test the object’s behavior before any changes are implemented in the physical object.

Another issue that should be considered in projects that use AMR or AGV is safety. When AMR or AGV are deployed in industrial or commercial settings, certain machine safety requirements must be met. In the European Union, all machines offered on the market must comply with the Machinery Directive [33] and are subject to risk assessment pursuant to EN ISO 12100: 2021 [34]. Any vehicle which is designed to operate in an industrial setting is subject to ISO 3691-4: 2020, depending on its intended use [35]. For robots working in commercial, non-industrial environments (e.g., an office building), the requirements are set out in ISO 13482: 2014 [36]. In geographic areas outside Europe, the safety requirements for machinery are provided for by separate national regulations, e.g., the American Occupational Safety and Health Administration (OSHA) regulation.

Both ISO 3691-4: 2020 and ISO 13482: 2014 set a number of requirements that an AMR must meet. These regard

- the maximum speeds at which an AMR can travel,
- the AMR’s ability to identify the operating zone in which it is located (and adjust its behavior to this operating zone, e.g., by measuring its own speed or changing the direction of travel),
- the vehicle’s behavior when a human or another safety-related object is detected), e.g., sending a visual warning signal, changing direction of travel,
- maximum braking distances and the maximum pressure forces that the robot can exert on a human or another safety-related object,
- control of the driving direction, and
- the vehicle’s behavior during steering in manual mode and in service mode.

In addition, these standards specify a number of requirements for the robot’s operating space and how it must be adjusted to ensure the safety of operators, including:

- designation and marking of permanent communication paths,
- designation and marking of operating zones, areas which must not be entered by operators (e.g., fencing off the robot’s operating zone), and areas which must not be entered by vehicles.

Algorithms related to the safe operation of an autonomous vehicle can be implemented and tested in a digital twin environment. Similarly to navigation and localization algorithms, safety functions can also be assessed in a virtual environment. One advantage of this approach is that a hazardous situation (e.g., a man falling down in front of a moving robot) can be simulated and assessed in a virtual environment before the appropriate algorithms are transferred to the real object. Some cases of use, like the example given above, could be dangerous or impossible to test in a real-life setting. Additionally, problems related to the robot’s operating environment are worth modeling using the digital twin. By doing so, one can check different variants of the process and various configurations of the operating environment (e.g., warehouse layout options, new communication routes) before they are implemented in the real-life object, which significantly reduces the costs and the number of changes made to the physical object.

Once the project has reached full maturity and has entered the operational phase, after the prototyping and implementation phases, the digital twin can still be used as a very helpful tool. The purposes for which it will be used may, of course, change. An important issue is the operational strategy adopted for AGV or AMR [37]. Most commonly, these vehicles are equipped with diagnostic functions that monitor the operation of individual subassemblies. By monitoring motor temperature and currents, one can determine whether the power drive systems are working properly and whether dangerous anomalies may occur in them (e.g., as a result of overheating or overloading). Data collected from the robot’s internal sensors can also be used to predict potential failures or wear, which can be prevented by timely maintenance. Similarly, it is possible to monitor the operation of power supply systems or communication modules [38].

From the operator’s point of view, the greatest advantage of using a digital twin is the possibility of observing the vehicle’s operation in a virtual environment. This allows the operator to control the robot’s actions, send commands to the robot to perform logistics tasks, and monitor the work progress on these tasks. The benefits of using a digital environment scale up with the increase in the size of the vehicle fleet, especially when it is supervised by one operator only. When managing a fleet of robots, managerial staff can use the data collected by real objects and stored in the digital twin environment to produce statistics and calculate KPIs for the operation of the intralogistics system [39,40,41]. The archiving of data from real-world objects allows managers to obtain information on.

- the average number of logistics tasks one vehicle/robot performed per day,
- the average time a vehicle/robot performed one logistics task (and how that time compares to the planned operation time),
- how many of the tasks assigned to the vehicles required the operator’s manual intervention (e.g., the robot incorrectly estimated its position in the operating environment or was stopped due to a failure),
- the efficiency of using the robot,
- whether there have been any security incidents (e.g., the operator stepping in front of the moving vehicle, which necessitated a manual restart of the robot’s safety system, which is required in selected operating zones).

In addition, when the space in which the vehicle moves is represented in the digital twin, the operator can send driving commands to the robot. If a robot is coupled to an automatic logistics system that manages the operation of the entire fleet of vehicles, the operator may not know what instructions have been given to the individual robots. Representation of vehicles and their operating environment as digital twins and the integration of the twins with the automatic logistics system allows the operator to remote-control the operation of the entire fleet and take appropriate measures (whenever necessary). When the physical environment is represented in the digital space, there is no need for the operator to be physically present near the vehicles’ workspace.

The concept of the digital twin can be applied at various phases of a project to provide a number of options and benefits (as listed in Table 1). At the stage of research and prototyping, a digital twin allows to prototype and develop a product without access to the physical object; during operation in the target physical environment, it allows for supervision of operations, the sending of commands, and controlling of the operating parameters; from the point of view of managerial staff responsible for logistics processes, a digital twin provides access to collected data, which can be used for efficiency analysis and reliability prediction. Considering all these aspects, it seems reasonable to use the methods and capabilities offered by digital twins in projects making use of AMR robots. The benefits of using a digital environment scale up as the complexity of the overall system and the size of the vehicle fleet increase. In the next section, we present an example of the application of a digital twin in an R&D project involving an AMR vehicle designed to execute internal logistics operations in a production company.

## 4. Case Study

For AGV or AMR to be implemented in a company, they need to meet a number of requirements. In the previous section, we listed problems to which the use of digital twins could provide some promising solutions. One of these problems, namely “Testing and validation of navigation algorithms in different vehicle operating conditions”, seems to be particularly interesting from the point of view of implementation This problem is of interest to both suppliers and end users of digital twins. However, it is particularly important to companies whose shop floors have not been designed to accommodate mobile robots. Production halls often contain infrastructure elements (poles, ramps, stairs) which may make it difficult or impossible for a robot to move around, be it due to its shape or the type of its drive system kinematics (e.g., a non-holonomic drive). Modifications to shop floor infrastructure create additional implementation costs and sometimes may be infeasible (e.g., structural elements of a factory building cannot be removed). Therefore, before a company purchases autonomous vehicles, or even when it is already implementing an autonomous vehicle technology, an appropriate trial/test environment is needed, which will allow the technology to be tested from the perspective of the company’s specific conditions.

A traditional solution to such a problem would be to conduct a series of tests in which different, but also repeatable, operating conditions would have to be recreated or set up using the real object. However, it is not always possible to perform tests in real-world settings. Additionally, such tests require the engagement of the company and its employees. In extreme cases, tests can lead to difficulties that may even halt production.

For these reasons, solutions alternative to real-life testing are sought, and this is where the digital twin is useful. Digital twins of AMR and their target operating environments can be constructed to test, in virtual reality, the robots’ control and navigation algorithms and to check whether they are performing the planned intralogistics tasks correctly. Simulations and analyses conducted using a digital twin allow the evaluation of a system’s behavior in various scenarios, especially atypical ones. This approach makes it possible to meticulously configure the robots, adjusting them and their workspace to the specific shop floor conditions and the intralogistics tasks the robots will be performing. In this way, the implementation time is reduced as the solution does not have to be tested, or is only partly tested, in real-life conditions. The digital twin technology is thus a standard solution that can be tailored to the needs of a specific company. Of course, final testing should be carried out in the actual production environment, but it should be kept to a minimum.

In this paper, we conducted a case study to check whether the use of a digital twin does bring the expected benefits. The findings are discussed in the sections below.

### 4.1. Goal

The concept of a digital twin was used to verify the design assumptions at an early phase of implementing an autonomous AMR vehicle in a company’s production hall. Before trials with the actual robot were conducted on the shop floor, digital models (digital twins) of the vehicle and the shop floor along with its equipment were created. Then, numerous computer simulations were carried out to test, assess, and improve the robot’s localization and navigation algorithms and travel paths, execution times for the planned intralogistics tasks, etc.

The following sections provide short descriptions of the vehicle, its target operating environment, the digital twins created for them, and the simulation software used. Then, a case of verification and improvement of a selected intralogistics task executed in a potentially “problematic” part of the shop floor is described.

### 4.2. The Autonomous Vehicle and Its Operating Environment

**Figure 4:**
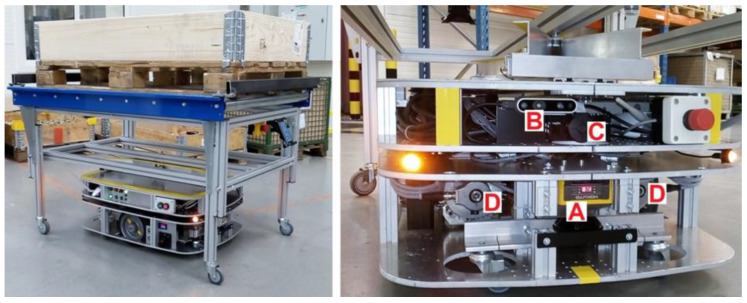
The prototype of autonomous mobile robot under the transport trolley (**left**). Main sensors of the robot (**right**): A—safety laser scanner, B—stereo vision 3D camera, C—high definition 2D camera, D—motor incremental encoders.

The main component of the automated transport system being implemented was the prototype of the AMR type transport vehicle shown in Figure 4. The robot towed overhead a rack (trolley) with a load placed on an EUR-pallet or in a box-pallet. The vehicle had two independently powered wheels which moved in a single axis (a differential system with (2.0) kinematics) and self-aligning castor wheels.

The robot was equipped with ambient sensors and an on-board control system, which allowed it to

- determine its position and orientation in the global reference system,
- autonomously plan the travel path to a given destination point,
- follow the planned path, and to detect and safely avoid obstacles along its way.

**Figure 5:**
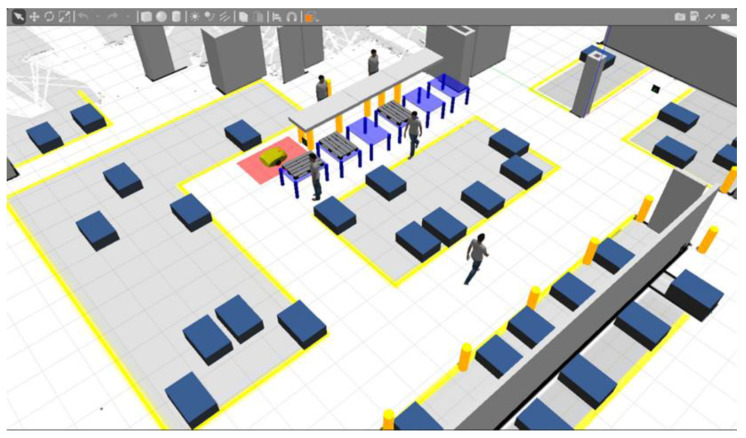
View of the digital twin of the real factory hall (model created in Gazebo simulator).

The robot’s task was to automatically execute the intralogistics tasks assigned to it on the shop floor, a part of whose digital model is shown in Figure 5. The AMR could only move through safe corridors between the areas marked with the yellow line on the ground (storage fields).

The Adaptive Monte Carlo Localization (AMCL) algorithm [26] was used to allow the vehicle to localize on the basis of information from its safety laser scanners. A stereoscopic camera had been installed at the front end to detect objects below and above the detection plane of the laser scanners. The vehicle’s motion path was planned using Search-based Planning with Motion Primitives, as described in [42] and implemented in the software package Search-Based Planning Library.

### 4.3. Digital Twins

Computer simulations with the digital twins of the AMR vehicle and the production hall were carried out using *Gazebo* software [43]. *Gazebo* is an environment dedicated to the simulation and high-quality visualization of models of robotic systems and their surroundings (workspace). *Gazebo* has a computing engine (numerical procedures) that simulates the physical phenomena occurring in mechanical systems, including the kinematics and dynamics of rigid bodies (also in movable joints), interactions via contact, friction, and gravity, and even the influence of wind. Importantly, it permits the simulation of the operation of virtual counterparts of sensors used in robotics, such as accelerometers and gyroscopes (IMU), distance scanners, and digital (2D) and stereoscopic (3D) cameras.

Figure 5 shows a visualization of a fragment of the digital twin of the shop floor created in the *Gazebo* simulation environment. The model incorporates fixed elements (walls, columns, racks, roller conveyors), box-pallets in storage areas, and the figures of employees. These components are important from the point of view of the operation of the simulated vehicle’s sensors and the robot’s localization and navigation algorithms.

**Figure 6:**
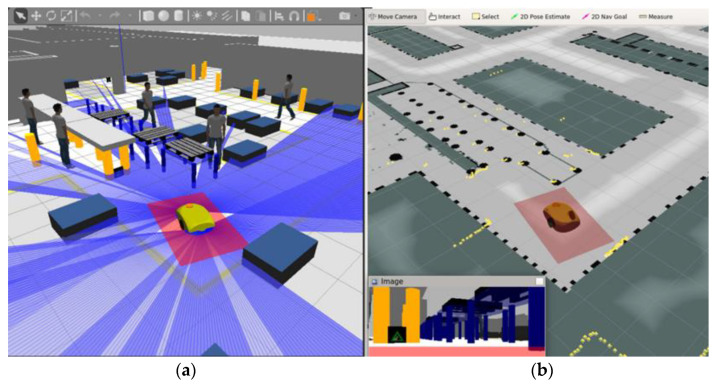
View of digital twin of real AMR in Gazebo simulator (**a**). Visualization of estimated AMR pose on the occupancy grid of production hall (**b**).

Figure 6a shows the digital twin model of the AMR (without the transport trolley) positioned on the floor of the virtual factory hall. The blue rays parallel to the floor around the vehicle visualize the operation of two simulated laser distance scanners (installed at the front and at the back of the robot). The red rectangle underneath the vehicle marks the protected area monitored by its security scanners.

Figure 6b shows a window of the *RViz* program designed for visualization and monitoring of the operation of robotic systems and their control algorithms. *RViz* is part of the *Robot Operating System* (*ROS*) middleware suite [44]. The window shows the model of the AMR against the background of an occupancy grid—the areas in the plane of the shop floor shown as a grayscale image. The black pixels on the occupancy grid indicate the forbidden zones, which the vehicle cannot enter. Grid pixels with a shade closer to white will be preferred by the navigation algorithms during path planning.

Yellow dots in Figure 6b visualize the results of measurements of the distance from objects detected by the robot’s digital twin’s virtual laser scanners. The digital model of the laser distance meters takes into account the random nature of the variability of the measurement results (characterized by a given normal distribution). It should be emphasized that the vehicle’s position and orientation on the occupancy grid (i.e., in the global reference frame of the shop floor—Figure 6b) are estimated by the AMCL algorithm and may therefore be burdened with error. In the bottom left corner of the *RViz* window, one can also see the image of the vehicle’s operating environment from a simulated camera installed on the front end of the robot’s digital twin.

The vehicle control, localization, and navigation algorithms exchange information with the real robot’s sensors and drive controllers via communication mechanisms implemented in *ROS*. The *Gazebo* simulator, in which the digital twins of the robot and the shop floor are represented, is also compatible with these mechanisms, which means the real robot and its sensors can be very quickly replaced with digital twins simulated in *Gazebo*.

**Figure 7:**
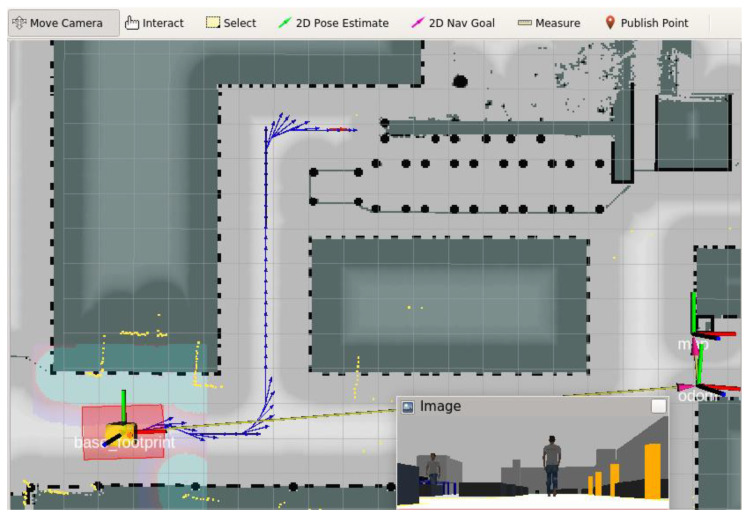
Visualization of calculated path for AMR to the dock position (at the top), blue arrows show subsequent desired poses of AMR.

Figure 7 shows the results of the operation of the algorithm planning the robot’s path from the current pose to the goal pose (at the top of the window). The blue arrows represent the poses set for the vehicle in the successive stages of its journey. All the algorithms of the robot’s on-board control system worked with digital twins (image from a simulated camera is shown) in the same way as they would have worked with the real vehicle in the real factory hall.

### 4.4. Improvement of the Intralogistics Task

**Figure 8:**
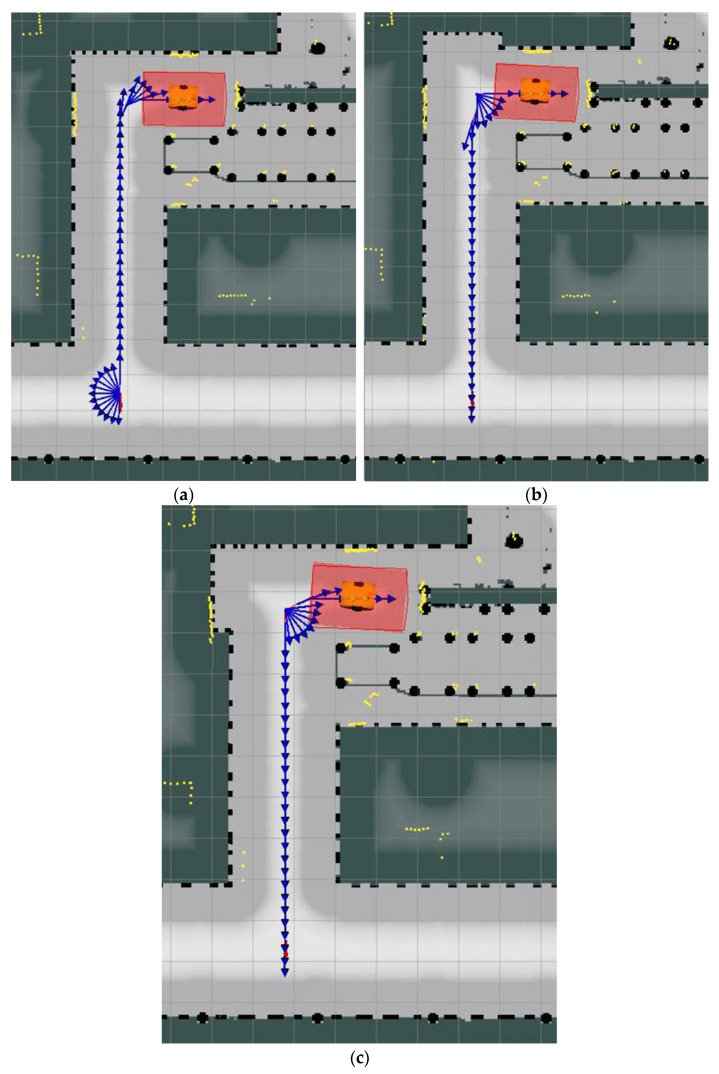
Calculated paths for AMR starting from the dock position (at the top) to destination pose (at the bottom) on the original occupancy grid (**a**) and on modified occupancy grid (**b**,**c**).

The digital twins of the AMR and its operating environment described in this paper were used extensively during the development, improvement and testing of the robot’s on-board localization and navigation systems. Figure 7 shows the path, planned by the navigation algorithm, leading to the docking station located at the end of the roller conveyor (at the top of the figure). The path runs along a safe corridor between prohibited areas which the robot is not allowed to move in. The simulations demonstrated that, despite the fact that the corridor in which the robot was allowed to move was relatively narrow relative to the size of the protected zone around the vehicle, the robot did reach the given docking position. However, the next simulations showed that the robot could only complete the return path by driving backwards—Figure 8a (here, the vehicle is in its initial pose) even though the planning algorithm preferred driving in the forward direction. Unfortunately, the corridor was too narrow for the robot to turn around on the spot.

In addition to the front and rear safety laser scanners, the prototype robot was equipped with a stereo vision (3D) camera and a high-resolution (2D) camera, which were mounted only at the front of the vehicle—Figure 4. The 3D camera allowed the robot to detect objects in front of it, also those “invisible” to the front security scanner (which operated only in one plane, parallel to the ground, Figure 6a). On the other hand, the 2D camera and the automatic image analysis algorithm helped the robot to find the passive navigation markers placed in the factory hall. The fact that the robot could “see” better the surroundings in front of it was precisely why the robot’s control system preferred for the robot to move forward. Notice that the AMR’s maximum forward speed was 0.7 m/s, while its maximum backward speed was only 0.3 m/s.

We performed a series of simulations which showed that the vehicle completed the path presented in Figure 8a, driving in reverse only, over the average time t_A_ = 44 s. In view of the above, we proposed two variants of the robot’s safe corridor in which the forbidden zone was modified slightly by adding a 2 × 0.4 m rectangular recess visible in the upper part of Figure 8b,c. The vehicle could use this recess to turn around on the spot and then cover a large part of the return path at a higher speed driving forward (Figure 8b,c).

The average travel times in the series of simulations for the modified versions of the robot’s safe corridor (Figure 8b,c) were t_B_ = 22.5 s and t_C_ = 23.3 s, respectively, i.e., they were almost 50% shorter than for the initial version of the corridor (Figure 8a). Given that additional sensors were only installed on the robot’s front end, the risk of collision with obstacles was reduced since the vehicle completed most of its path driving forward. The results of the simulations are summarized in Table 2.

## 5. Conclusions

In this paper we showed how the Digital Twin technology could be used in the design and implementation of an AMR in a production hall that offered limited possibilities of planning the robot’s paths. Our investigations led to the following conclusions:

- The use of the Digital Twin technology accelerated the development of AMR localization and navigation algorithms under various operating conditions, at the same time reducing the costs of testing and validating them.
- The computer simulations, conducted with the use of a digital twin, allowed us to improve the shape of the AMR’s communication route. As a result, the time which the robot needed to complete its intralogistics task was reduced by half.
- It should be underlined that the scenarios we considered were specific for the given company. A solution was presented which required only a slight modification of the robot’s safe corridor.
- Not only the robot’s travel time, but also the time of system implementation was reduced. Testing was carried out remotely, without the need to perform on-site tests.
- The case study confirmed that the use of a digital twin brought the expected benefits.

The positive results we obtained provide motivation for further research into the potential benefits of using digital twins in other areas of technology and industry.

The significant problem that increased during the provided research on the digital twin and AMR vehicle modeling was the lack of uniform standard of design patterns. Moreover, there are no universal IT tolls, software, or frameworks that help to quickly and efficiently develop digital twins. In our opinion, this constraints can cause real difficulties when apply digital twins in real industrial conditions. The low level of universalism and lack of proven design patterns will provide the necessity of individual treatment of each implementation of digital twin in industry. Moreover, the design teams can be forced to develop their own IT tools and programming libraries, which can cause the increasing cost of digital twin implementation. What is more, the time of spreading such solutions in the commercial environment will be much longer. To summarize, it can be noticed that the conception of digital twins has been already well described in the literature and is well-known in many research and industrial centers. Unfortunately, there are still no necessary design patterns and universal IT tools that effectively support this area of Industry 4.0 in real industrial conditions and there is a strong necessity to carry out further works in this area.

## Figures and Tables

**Table 1:** Benefits of using digital twins of AMR/AGV and their operating environments.

Problem	How the Problem Is Solved without Using a Digital Twin	How the Problem Is Solved Using a Digital Twin	Benefits of Using a Digital Twin
Development of robot navigation and localization algorithms	Experiments and measurements are conducted using a real-world object	Experiments and measurements are conducted using a digital object	No need to access the physical facility.
			Algorithms can be worked on remotely.
			Work can be done in distributed teams
Testing and validation of navigation algorithms in different vehicle operating conditions	Different operating conditions are re-created/set up using the real-life object	Different operating conditions are defined on the digital object	The different operating conditions can be tested multiple times.
			Very rare/unique working conditions can be defined.
Testing and validation of safety functions	A potentially hazardous situation can be staged using the real-life object	A hazardous situation can be defined on the digital object	The behavior of the safety function can be tested in various hazardous situations (including extreme and very dangerous ones).
			The consequences of the occurrence of hazards can be analyzed
Collecting diagnostic data and predicting reliability	Measurements are made and diagnostic data are collected off-line in the vehicle’s internal memory.	Diagnostic data are collected online while the robot is in operation.	Access to online diagnostic data
	The vehicle’s reliability is analyzed post factum	The vehicle’s reliability is analyzed online	Failures can be predicted
Logistics commands	Commands are sent to individual robots.	Commands are sent to the entire fleet of vehicles.	A whole fleet of robots can be managed. One operator can oversee a larger number of vehicles.
	The logistics tasks of a single vehicle are controlled	The logistics tasks, operation and condition of the entire fleet are controlled collectively	The fleet’s operation can be managed remotely without managers actually being present physically in the factory/warehouse (remote fleet management)
Robot fleet management	Fleet performance data are collected manually	Fleet performance data are collected automatically	Fleet performance is determined automatically
			Multidimensional analysis of the fleet is possible as large amounts of data of various types (regarding reliability, logistics tasks, journeys, safety, etc.) are collected

**Table 2:** Simulated AMR travel times for three different shapes of the robot’s safe corridor.

Shape of the AMR’s Safe Corridor	Figure Number	Average Travel Time [s]	Relative Reduction in Travel Time after Modification of the Corridor [%]
Initial (before modification)	8a	44.0	−
With an additional recess “at the top”	8b	22.5	49
With an additional recess “on the left”	8c	23.3	47

## Data Availability

All the data is available within the manuscript.
